# Under the Digital Bridge: Investigating Trolling Behaviors in Baby Boomers

**DOI:** 10.1093/geroni/igaa057.1015

**Published:** 2020-12-16

**Authors:** Kristen Hardin-Sigler, Rebecca Deason, Stephanie Dailey, Natalie Ceballos, Krista Howard

**Affiliations:** Texas State University, San Marcos, Texas, United States

## Abstract

Internet trolling, or the intentional disturbance or upsetting of others on social media for personal amusement, has become increasingly prevalent in recent years (Howard et al., 2019). Current research focuses on these destructive social media behaviors in younger populations, therefore this study set out to investigate the gender differences of trolling behaviors in Baby Boomers. Participants (N = 140), ages 54 and older, were recruited from the Amazon Mechanical Turk and were compensated for their participation. Participants completed a survey investigating their likelihood to engage in trolling behaviors, the extent to which they enjoy trolling, and their feelings while trolling. Results indicated that while there were no significant differences between men and women in their need, intensity of use, or addiction to social media, men were significantly more likely to engage in trolling behaviors than women. Men reported posting to upset others (p = .018), as well as commenting to upset others (p = .053), more often than women. Furthermore, when engaging in these behaviors, men reported feeling intelligent (p = .013), confident (p = .024), superior (p = .053), and happy (p = .012), more often than women. However, these results could be indicative of a more sinister issue. Men also reported more often that their reasons for engaging in trolling behaviors were feelings of loneliness (p = .005) and anxiety (p = .010). This indicates that these trolling behaviors may then be a way for men to seek out some form of “social support” in the online community.

